# Is there any role for HBV pgRNA in fibrosis and HCC predisposition?

**DOI:** 10.3389/fmed.2025.1678116

**Published:** 2025-11-12

**Authors:** Aikaterini Skeva, Theocharis Konstantinidis, Vasileios Papadopoulos, Maria Panopoulou, Konstantinos Mimidis

**Affiliations:** 1 Laboratory of Microbiology, Department of Medicine, Faculty of Health Sciences, Democritus University of Thrace, Alexandroupolis, Greece; 2 Laboratory of Anatomy, Department of Medicine, Democritus University of Thrace, Alexandroupolis, Greece; 3 First Department of Internal Medicine, Democritus University of Thrace, Alexandroupolis, Greece; 4 Laboratory for the Study of Gastrointestinal System and Liver, Democritus University of Thrace, Alexandroupolis, Greece

**Keywords:** Chronic Hepatitis B, CccDNA, HBeAg negative, pregenomic RNA, surrogate marker, NAS, FIB-4, PAGE-B

## Abstract

**Aim:**

In this cohort, we aimed to study the evolution of pregenomic RNA (pgRNA) during treatment and compare it with other disease scores such as FIB-4 and PAGE-B.

**Methods:**

Eighty-eight HBeAg negative CHB who received long-term treatment with NAs were included. A quantitative HBV S antigen (HBsAg) assay was performed, and viral HBV DNA was quantified by Polymerase Chain Reaction (PCR). Finally, viral RNA levels (pre-core RNA (preC RNA) and pgRNA) were analyzed using the RTPCR protocol. The FIB-4 score was calculated for all patients, depicting the cirrhosis course, while the platelet-related PAGE-B score contributed to the 5-year cumulative prognosis of hepatocellular carcinoma (HCC). Statistical multivariate analysis was performed using the R studio and CATREG SPSS optimal scaling algorithm of SPSS 26.0.0.0.

**Results:**

A total of 18.1% of our sample was positive for HBV pgRNA, delineating a positive correlation with cirrhosis and an apparently negative correlation with therapy duration. HBV pgRNA was not independently correlated with FIB-4 (*p* = 0.137) after adjustment for aminotransferase/alanine transaminase (AST/ALT)^1/2^, (AST)^1/2^, 1/platelets (PLT), age, sex, HBsAg, HBV viral load, regimen administered, and therapy duration (ordinal regression ANOVA *p* < 10^−12^; ${\text{R}}_{\text{reg}}^{2}$: 0.794). Moreover, HBV pgRNA was not independently correlated with PAGE-B (*p* = 0.459) after adjustment for age, sex, AST, 1/PLT, duration of therapy, HBsAg, HBV viral load, regimen administered, and the presence of cirrhosis (ordinal regression ANOVA *p* < 10^−12^; ${\text{R}}_{\text{reg}}^{2}$: 0.800).

**Conclusions:**

Based on our results, further longitudinal studies are needed to assess the potential usefulness of HBV pgRNA as prognosticator of liver fibrosis and susceptibility to HCC.

## 1 Introduction

Hepatitis B virus (HBV) belongs to the family *Hepadnaviridae* and consists of an outer envelope, inner nucleocapsid, and 3.2kb relaxed circular DNA (RC-DNA). Approximately 240 million individuals worldwide have Chronic Hepatitis B (CHB). HBV is a mutagenic virus that causes almost 40% of untreated individuals to develop cirrhosis and hepatocellular carcinoma (HCC). Dane infectious particles are inserted into hepatocytes and form a mini-chromosome wrapped with histones, named covalently closed circular DNA (cccDNA) (1). There are four overlapping open reading frames (ORFs), one of which is HBV pgRNA, which serves as a template for reverse transcription while forming the mRNAs for polymerase and core protein transcription. The preC mRNA encodes the HBV e antigen (HBeAg); two different smaller transcripts assist in the formation of large, medium, and small envelope proteins, Hepatitis B S antigen (HBsAg), whereas the smallest of all transcripts encodes the regulatory X protein (HBx) (2–4).

Patients with chronic hepatitis B are more likely to develop cirrhosis and HCC. Usually, HBeAg-positive patients are at a higher risk of cirrhosis than HBeAg-negative patients, frequently leading to decompensated cirrhosis (5). FIB-4 is a cirrhosis prognosticator with a 95% specificity for CHB and HBeAg-negative patients. A cut-off value of 3.25 indicates those with advanced cirrhosis (F4; >3.25), while a cut-off of 1.45 excludes from cirrhosis incidence (< 1.45) (6). The advantage of this non-invasive score is that it is based on variables such as age, AST/ALT levels, and platelets, which can be easily collected in favor of the FIB-4 score (7). Moreover, PAGE-B is a non-invasive prognostic score that contributes to 5-year cumulative HCC incidence prediction among Caucasians. By combining easily accessible information, such as sex, age, and platelet count, CHB patients are categorized into three categories based on the risk incidence (8). Patients scoring ≤ 9, 10–17, ≥ 18 had 0%, 3%, and 17% 5-year cumulative HCC incidence, respectively. Although these prognostic scores effectively contribute to cirrhosis and HCC occurrence, they do not include molecular biomarkers that can personalize and improve the effectiveness of the scores.

Virological relapse and clinical relapse are present, especially in HBeAg-negative patients, where although cccDNA levels are drastically reduced, HBsAg is still present (9). In parallel with possible virological or clinical relapse, there is a high incidence of cirrhosis and HCC, which can be predicted using a surrogate marker that shows the transcriptional status of cccDNA. HBV pgRNA seems to be a highly promised biomarker that could be easily detected in serum and detailly describes the hepatocellular cccDNA status. Given that NA therapy does not act on cccDNA replication there are cases where, although the HBV DNA is absent and the HBs Ag seroconversion has been reached, the cccDNA is present in hepatocytes without a functional cure to be made (10). Furthermore, studies show that patients undergoing NAs discontinuation after an HBs Ag seroconversion can easily face illness relapse, due to the presence of hepatocellular cccDNA and HBV pgRNA in the circulation (11). Significant findings depict the independent correlation of HBs Ag low levels with the presence of HBV pgRNA by unveiling the positive correlation between the two markers (12). For example, the levels of HBV pgRNA can specifically demonstrate the HBs Ag levels and suggest, either the NAs discontinuation or the liver inflammation status in HBe Ag-negative patients under NA therapy (13).

More specifically, to date, only HBsAg seroconversion has been considered a good reason for NAs discontinuation, while except from pgRNA also HBV core-related antigens have also been studied as possible treatment discontinuation biomarker (14, 15). Laras et al. (16) contributing to the research on an alternative NAs discontinuation marker, suggested that pgRNA-positive patients after NAs discontinuation could not achieve HBsAg clearance, opposed to pgRNA-negative patients. Moreover, the presence of circular pgRNA seems to be a good predictor for the liver inflammation status, because it regulates pathways related to cancer stemness and progression (17, 18). However, its performance as an independent biomarker is still poor and more studies must be conducted to enhance its dominance as a valuable fibrotic or malignant index.

In this study, the HBV pgRNA levels of Caucasian individuals receiving entecavir/tenofovir were correlated with FIB-4 and PAGE-B scores. The main aim was to predict the transcriptional activity of cccDNA and delineate how pgRNA levels contribute to the predictive value of the known FIB-4 and PAGE-B scores in the Caucasian population (8, 19).

## 2 Materials and methods

### 2.1 Patients and biological samples

In this study, we examined 88 plasma samples collected between January 2022 and August 2023 in the hepatology outpatient clinic of the First Department of Internal Medicine. Each sample belonged to a patient with CHB hepatitis who was under NA treatment for at least 1 year (Table 1). The clinical status of our patients was clear from any cirrhotic or malignant incidence before the treatment initiation, all of them were tested with elastography for the liver inflamation status. The patients started their therapy after tested for ALT levels (min-max: 20–54 IU/ml; median: 31 IU/ml), HBV DNA (median: 2.2^*^10^5^IU/ml) and HBs Ag (min-max; 45–5,562 IU/ml; median: 324.2 IU/ml), while no HBV pgRNA measurements are present for this illness stage. Patients with HCC as well as patients with concomitant infections by Hepatitis D Virus, Hepatitis C Virus and Human Immunodeficiency Virus were excluded. All patients were Caucasian and examined for the HBV genotype, which was predominantly found to be positive for the D type. All plasma samples were stored in aliquots at −80 °C and thawed once for analysis. All our patients were under NA treatment, with some showing a detectable HBV DNA load either due to early therapy initiation or due to no response to NAs therapy. There were 22 HBV DNA load-positive individuals (Table 1).

**Table 1:** Patients' characteristics according to HBV pgRNA status.

**Variables**	**Overall (*n* = 88)**	**HBV pgRNA negative (*n* = 72)**	**HBV pgRNA positive (*n* = 16)**	***p-value***
**Age**				
Years (mean ± SD)	58.6 ± 12.4	60.8 ± 11.4	49.0 ± 12.2	< 0.001
**Gender**				
Male	43 (49)	35 (49)	8 (50)	0.920
Female	45 (51)	37 (51)	8 (50)	
**AST**				
U/L; median (IQR)	24 (19–31)	22 (18–27)	33 (25–39)	0.001^†^
**(AST)** ^1/2^				
median (IQR)	4.8 (4.4–5.5)	4.7 (4.2–5.2)	5.7 (5.0–6.2)	0.001^†^
**ALT**				
U/L; median (IQR)	22(17–34)	22 (16–31)	36 (25–55)	0.001^†^
**(1/ALT)** ^1/2^				
(L)^1/2^; median (IQR)	0.21 (0.17–0.24)	0.22 (0.18–0.25)	0.17 (0.14–0.20)	0.001^†^
**AST/ALT**				
median (IQR)	1.0 (0.8–1.3)	1.0 (0.8–1.3)	0.9 (0.9–1.7)	0.854^†^
**(AST/ALT)** ^1/2^				
median (IQR)	1.0 (0.9–1.1)	1.0 (0.9–1.1)	1.0 (0.9–1.3)	0.854^†^
**PLT**				
10^9^/L; median (IQR)	222 (162–239)	204 (164–236)	231 (132–247)	0.665^†^
**1/PLT**				
μL; median (IQR)	4.45 (3.89–5.64)	4.53 (3.96–5.65)	4.12 (3.46–5.14)	0.215^†^
**HBsAg**				
median (IQR)	522 (56–3,003)	309 (39–2,337)	3,003 (319–5,446)	0.002^†^
**HBV viral load**				
copies/ml; median (IQR)	0 (0–21)	0 (0–0)	783 (0–2,968)	< 0.001^†^
**HBV viral load presence**				
No	66 (75)	61 (85)	5 (31)	< 0.001^‡^
Yes	22 (25)	11 (15)	11 (69)	
**HBV pgRNA**				
**copies/ml; median (IQR)**	1.4 (1.0–1.9)	0 (0–0)	776 (564– 2,018)	< 0.001^†^
**Regimen**				
ETV	50 (57)	39 (54)	11 (69)	0.287
TDF	38 (43)	33 (46)	5 (31)	
**Duration of therapy**				
months; median (IQR)	114 (39–168)	144 (60–168)	18 (5–72)	< 0.001^†^
**Therapy** > **6 years**				
No	37 (42)	24 (33)	13 (81)	0.001^‡^
Yes	51 (58)	48 (67)	3 (19)	
**FIB-4 score**				
median (IQR)	1.3 (1.0–1.9)	1.3 (1.0–1.9)	1.1 (0.9–2.2)	0.566^†^
**Advanced fibrosis (METAVIR stage F3/4) likely (FIB-4 score** ≥**2.67)**				
No	79 (90)	66 (92)	13 (81)	0.355^‡^
Yes	9 (10)	6 (8)	3 (19)	
**Approximate Ishak fibrosis stage**				
0–1	53 (60)	43 (60)	10 (62)	0.066^‡^
2–3	29 (33)	26 (36)	3 (19)	
4–6	6 (7)	3 (4)	3 (19)	
**Cirrhosis (Fibroscan documented)**				
No/missing	75 (85)	62 (86)	13 (81)	0.698^‡^
Yes	13 (15)	10 (14)	3 (19)	
**PAGE-B**				
median (IQR)	12 (8–16)	12 (10–16)	9 (5–14)	0.459^†^
**PAGE-B stage**				
1 (≤9)	26 (30)	21 (29)	5 (31)	0.979
2 (10–17)	46 (52)	38 (53)	8 (50)	
3 (≥18)	16 (18)	13 (18)	3 (19)	

^†^Independent-Samples Mann-Whitney U test.

^‡^Fisher's exact test.

AST, Aminotransferase; ALT, Alanine transaminase; PLT, platelets.

### 2.2 Laboratory measurements

The biochemical markers ALT/AST, TBIL, DBIL, total protein, albumin, globulin, prothrombin time, and INR were measured, if available, for the collected samples. The measurement of biochemical markers: ALT/AST, TBIL, DBIL, total protein, and albumin, were performed by a fully automated, high throughput photometric analyzer (Roche Diagnostics GmbH, Mannheim). More specifically, AST/ALT quantitative analysis was determined in conjunction with pyridoxal phosphate activation in human serum and plasma (Roche Diagnostics GmbH, Mannheim). TBIL, DBIL, total protein, and albumin protein were measured by the colorimetric assay (Roche Diagnostics GmbH, Mannheim). Serum globulin's level was measured with electrochemiluminescence immunoassay “ECLIA,” (Roche Diagnostics GmbH, Mannheim). Coagulation profile of study population was performed by measuring prothrombin time and INR following activation of blood coagulation (ACLTOP, Werfen, San Diego, California).

Serological markers of HBV infection were measured by the qualitative chemiluminescent microparticle immunoassay (Alinity, Abbott, Ilinnois, US). Analytically: for the quantitative measurment of Hepatitis B surface antigen (HBsAg) in patients' serum, samples, anti-HBs coated microparticles, as well as antibodies, antiHBs acridinum-labeled conjugate were combined. After incubation, HBsAg binds to anti-HBs antibodies to form complex. The resulting chemiluminescent reaction was measured as relative light units (RLU). The same method was used for HBeAg and anti-HBe quantification.

The HBV DNA load was defined by RT-PCR using Roche 4800cobas X/Z, where a lower quantification limit was formed at 10 IU/ml (Minimum Titer) of plasma. Finally, all plasma samples were examined for HBV genotype using qualitative RT-PCR (Sacace, Como, Italy), and for the presence of HBV RNA.

### 2.3 Real time PCR for HBV genotype determination

All samples were tested for HBV genotypes based on qualitative determination by real-time PCR. Considering the distinct geographical domination of HBV genotype D in the Mediterranean region, we tested all our samples using the HBV genotype A-D real-time kit (Sacace, Como, Italy) (20).

### 2.4 Real time PCR set up for HBV RNA quantification

For HBV RNA quantification, we used the quantitative method of Laras et al. (16) using three pre-defined pairs of primers for the detection of preC mRNA, CP-directed transcript (preC mRNA plus pgRNA), and DNA contamination.

RNA was extracted from 400 ul plasma using a NucleoSpin Virus DNA/RNA kit (Macherey Nagel GmbH and Co, Düren Germany) and 10 ul eluate was pretreated for possible gDNA contamination and reverse transcribed using the PrimeScript RT Reagent Kit with gDNA Eraser (Takara Bio, CA 91043, USA). We used 5 ul eluate for the reverse transcription reaction and cDNA synthesis using the antisense BC1 primer (5′-GGAAAGAAGTCAGAA GGCAA, nt1974-1955). Then 1 ul from cDNA reaction was used for each of the three different RT-PCR reactions with BC1 as the common 3′ primer, PCP (5′-GGTCTGCGCACCAGCACC, nt1796-1813) as the 5′ primer for preC mRNA detection, PGP (5′-CACCTCTGCCTAATCATC, nt1826-1843) as the 5′ primer for CP-directed transcription, and M3 (5′-CTGGGAGGAGTTGGGGGAGGAGATT, nt1730-1754) as the 5′ primer for DNA contamination. The reaction mix was composed of MgCl 0.32 mM, forward/reverse primer (0.5 mM), probeFL (4 mM), probe LC (8 mM) (Supplementary Table 1), H2O, and Taq Polymerase/dNTPs solution from the LightCycler^®^ FastStart DNA Master HybProbe (Roche Diagnostics GmbH, Mannheim). The pgRNA levels were calculated by subtracting preC mRNA from the total CP-directed transcript for each sample, and DNA contamination was detected by examining the samples for CP-directed transcription and/or cDNA products.

To standardize the precision of the assay, we used sequentially diluted samples of a customized cloned plasmid containing the desired nucleotide sequence (Supplementary Table 2). After standardizing the assay, we determined the lower limit of detection (LLD) at 262 (2.42 log10) copies per ml (c/ml) and the LQD at 322 (2.51 log10) copies per ml (c/ml) serum.

### 2.5 Variable editing and grouping

pgRNA, HBV DNA, and HBsAg levels: Samples were deemed pgRNA-positive when the load was above the Lower Limit of Detection (LLD), 262 copies/ml. All other samples were treated as pgRNA-negative. The LLD for the HBV DNA load was 10 IU/ml. For HBsAg levels, LLD was 0.2 ng/ml.

HBeAg: All samples were tested for HBeAg positivity, and none were found to be positive for the e antigen.

Therapy duration and treatment: We considered the starting point of the treatment on the first day when our patients tested positive for HBV DNA, with levels exceeding 2,000 IU/ml. All the patients were treated with either entecavir or tenofovir NAs.

HBV genotyping: All samples were tested for one of the four HBV genotypes (A–D), all of which were positive for genotype D. Given the geographical distribution of HBV genotypes, the results indicated the prevalence of subtype D in the Mediterranean region. Genotypes A, B, and C are the next most common in Europe; therefore, we did not examine our patients for 10 different HBV genotypes (20).

### 2.6 Calculation of PAGE-B and FIB-4 scores

The PAGE-B score includes platelet count, age, and sex. Scoring was performed as follows: (i) For age 16–29 years, 0 points; 30–39 years, 2 points; 40–49 years, 4 points; 50–59 years, 6 points; 60–69 years, 8 points; ≥ 70 years, 10 points; (ii) for female sex, 0 points; for male sex, 6 points; and (iii) for platelet count (/mm3) ≥ 200,000, 0 points; 100,000–199,999, 6 points; < 100,000, 9 points. The scores ranged from to 0–25 points. Based on their PAGE-B scores, patients were classified as ≤ 9, low-risk; 10–17, moderate risk; and ≥18, high-risk Papatheodoridis et al. (9).

The FIB-4 score is also age-dependent, but also consists of ALT, AST, and platelet counts. The formula for FIB-4 is: (Age [yr] × AST [U/L])/((PLT [109/L]) × ($\sqrt{\text{ALT}}$ [U/L])) = Age*AST/(PLT*$\sqrt{\text{ALT}}$), and the score should be interpreted cautiously for ages below 35 (< 35) and above 65 (>65) years (14). FIB-4 efficiently calculates absence of advanced fibrosis for values below < 1.45 (F0–F1) and advanced fibrosis for values above >3.25 (F4–F6).

### 2.7 Statistical analysis

Continuous variables were expressed as means and standard deviations (SD) in cases where equal variances could be assumed and compared using the Student's *t-test*. In contrast, when the Levene test yielded a statistically significant result, the Mann-Whitney *U*-test was used to compare the medians and interquartile ranges (IQR). Discrete variables were expressed as percentages and compared using the chi-square test; in the case of expected frequencies < 5 in ≥25% of cells, Fisher's exact test was applied. The measure of the association between two binary variables was approached using the phi (ϕ) coefficient.

The most parsimonious multivariable categorical regression models were constructed to assess FIB-4 and PAGE-B scores as potential prognosticators using optimal scaling after maximal discretization (up to seven categories), ridge regression, and 10x cross-validation. To avoid multicollinearity, the minimum tolerance was set to 0.5. Kaplan-Meier curves were used to depict the cumulative probability of detection of HBV pgRNA vs. duration of therapy.

The level of statistical significance was set to 0.05. All tests were performed using SPSS 26.0, R (version 4.4.1) and GraphPad Prism version 8.

## 3 Results

All patients belonged to the Delta genotype and, therefore, were negative for HBeAg, but positive for anti HBe antibody. HBV pgRNA was detectable in 16 out of 88 samples (18.1%) with an average concentration of 776,25 c/ml (2,89 log10). All individuals were negative for a small fraction of CP-directed region (preC RNA).

Comparing the pgRNA-negative and pgRNA-positive groups there are distinguished differences for variables like, age (*p* < 0.001), ALT and AST levels (0.001), HBsAg levels (*p* = 0.002), HBV viral load (*p* < 0.001) and duration of the therapy (*p* < 0.001). Further investigation of the fitting between the above variables and the two scores FIB-4 and PAGE-B depicted a significant cirrhosis incidence for those with FIB-4 above 2.7; however, no significant determination was depicted for the PAGE-B score depending on the examined factors (Table 1).

### 3.1 HBV pgRNA and HBV DNA

Of the 88 patients under NA treatment, we observed 66 patients with a complete response to therapy (HBV DNA load = 0 IU/ml) and 22 patients with an incomplete response and detectable HBV DNA levels (< 20,000 IU/ml). The HBV DNA load variance was due to the treatment duration. More specifically, subjects with detectable HBV DNA load had a median treatment duration of 22.54 months, whereas patients with zero HBV DNA levels had 137.77 months under NA treatment (*p* < 0.0001). As a result, there were subjects who could not achieve HBV DNA clearance because of short-term NA treatment. Between the two categories, pgRNA positivity demonstrated a statistically significant difference, and the number of patients positive for HBV pgRNA was 31% in the HBV DNA-negative group and 69% in the HBV DNA-positive group (*p* < 0.001). In addition, the mean HBV DNA values seemed to be significantly different, as the HBV pgRNA-negative group had zero HBV DNA levels compared to the HBV pgRNA-positive group (0.00 vs. 783.0, *p* < 0.001).

HBV DNA load increases in parallel with pgRNA levels, and there is great statistical significance between detectable and undetectable HBV DNA loads. The ability to detect HBV pgRNA is more sensitive than that of HBV DNA load (18.1% vs. 34.0%) and shows the transcriptional persistence of the virus. When the two markers were positive, we observed the highest levels of HBV pgRNA, and the mean values between the two categories showed significant variance (HBV negative vs. HBV positive; 1.84 vs. 2.8, *p* = 0.0056) log10 copies/ml.

### 3.2 HBV pgRNA and HBsAg levels

HBsAg levels are the first sign of HBV infection, and usually, a decline in HBsAg levels does not follow long-term treatment. HBeAg-negative patients have low HBsAg levels; however only 1%−3% meet the seroconversion and HBsAg clearance criteria according to the EASL (21). HBV pgRNA-negative individuals had significantly lower mean HBsAg levels than HBV pgRNA-positive individuals (309 vs. 3,003; *p* = 0.002). Although all of our individuals were positive for HBsAg, we found that the pgRNA positivity rate followed the highest HBsAg levels. There was a fluctuation in the HBsAg levels between the two groups.

### 3.3 HBV pgRNA and therapy duration

A greater long-term therapy duration was noted in HBV pgRNA-negative individuals (144 months, *p* < 0.001), while HBV pgRNA-positive subjects were registered in the study with a median value of 18 months (*p* < 0.001). Dividing the therapy duration into two stages by applying a 6-year cut-off, we identified an increased percentage of HBV pgRNA-positive individuals in the group that was less than a year under NA therapy (HBV pgRNA-positive vs. HBV pgRNA-negative; 81% vs. 33%, *p* = 0.001). The second midterm included individuals under NA therapy for more than 6 years and represented a great percentage of negative HBV pgRNA individuals (HBV pgRNA negative vs. HBV pgRNA positive; 67% vs. 19%, *p* = 0.001).

**Figure 1:**
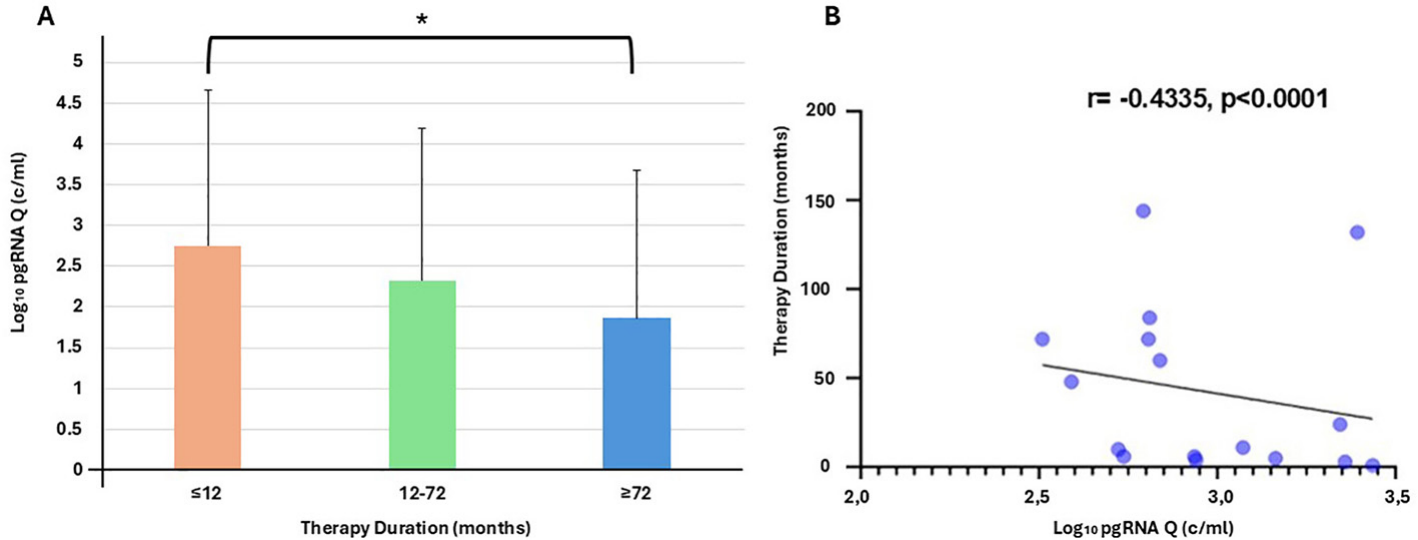
**(A)** Relation between therapy duration stages and pgRNA Q; ≤12 months vs. 12–72 months, (*p-value* = 0.058 > 0.05), 12–72 months vs. ≥ 72 months (*p-value* = 0.577 > 0.05), ≤12 months vs. ≥ 72 months (*p-value* = 0.021 < 0.05*), **(B)** Correlation of Therapy duration to the pgRNA quantitative levels in serum, depicts a strong negative assumption about the decline of pgRNA levels as the years of therapy are being increased, Spearman *r* = −0.4335, *p* < 0.0001.

In addition, we observed a significant difference in the median HBV DNA load values between the two groups (< 6 years under NA vs. >6 years under NA; 169 IU/ml vs. 0 IU/ml, p < 0.001). Adhering to these findings, the median HBV pgRNA levels showed a significant decline that followed the duration of therapy. In particular, we separated the therapy duration into three stages (≤12 months, 12–72 months, ≥72 months), and a visible gradient decrease in pgRNA levels was demonstrated. The pgRNA median values depicted a significant difference between the first and the last group (≤12 and ≥72 months, 614.30 vs. 73.17, *p* = 0.021, Figure 1A), showing the NA contribution in the therapeutic procedure.

Another parameter tested in this study was the correlation between therapy duration and HBV RNA level. This correlation showed a negative linear relationship between the two variables (Spearman *r* = −*0.4335, p* < 0.0001) (Figure 1B). In parallel, there was a positive linear correlation with pgRNA quantity for the following univariable correlations: ALT, HBV load, and HBsAg (Spearman *r* = 0.3726, *p* = 0.0004 < 0.001, Spearman *r* = 0.5114, *p* < 0.0001, Spearman *r* = *0.3464, p* = 0.0009 < 0.001), which opposes the years of treatment. Therapy duration had a negative linear correlation for each of the three variables. Futhermore, the two different therapuetic approaches either tenofovir or entacavir does not seem to differentiate our patients clinical status neither correlated with the presence or absence of HBV pgRNA (*p* = 0.287).

**Figure 2:**
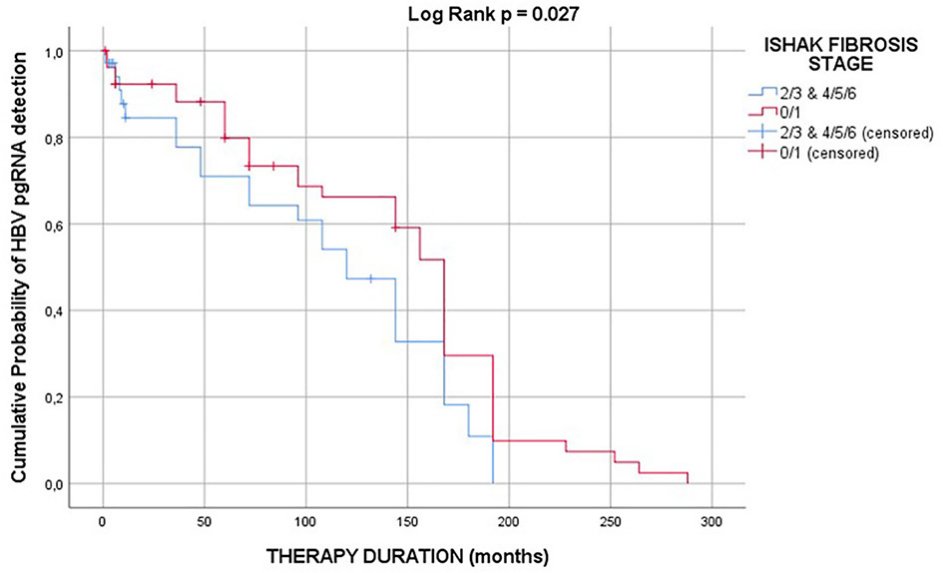
Kaplan-Meier curves depicting the cumulative probability of detection of HBV pgRNA vs. duration of therapy; detection of HBV pgRNA is prolonged (*p* = 0.027) in patients with Ishak fibrosis stage 0–1 (median HBV pgRNA survival 168 months; 95% CI: 157–179), when compared with patients with Ishak fibrosis stage 2–3 and 4–6 (median HBV pgRNA survival 120 months; 95% CI: 89–151).

Kaplan-Meier curves were constructed to depict the cumulative probability of detection of HBV pgRNA vs. duration of therapy; detection of HBV pgRNA was prolonged (*p* = 0.027) in patients with Ishak fibrosis stage 0–1 (median HBV pgRNA survival 168 months; 95% CI: 157–179), when compared with patients with Ishak fibrosis stage 2–3 and 4–6 (median HBV pgRNA survival 120 months; 95% CI: 89–151) (Figure 2).

### 3.4 HBV pgRNA and FIB-4

The assessment of advanced fibrosis, as assessed using FIB-4, was correlated with the presence of cirrhosis, as documented by FibroScan measurements (phi = 0.494; *p* = 4x10-6).

Moreover, the use of the FIB-4 score to assess FibroScan documented cirrhosis (AUROC curve: 0.783 ± 0.057; 95 CI: 0.671–0.895; *p* = 8x10-7; overall model quality: 0.67 (Supplementary Figure 1).

**Figure 3:**
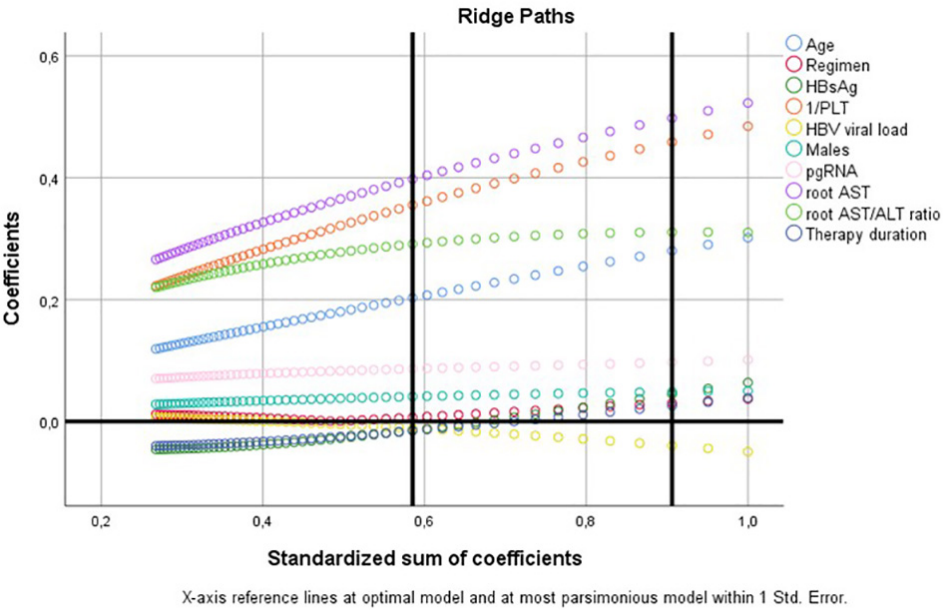
Categorical regression model assessing the potential value of HBV pgRNA as prognosticator of FIB-4 (Table 2); ridge paths.

To further investigate the potential role of HBV pgRNA title as an independent prognosticator for the FIB-4 score, we implemented a multivariate categorical regression model incorporating the latter as the dependent variable and age, (AST/ALT)1/2, (AST)1/2, 1/PLT, HBV pgRNA, male sex, duration of therapy, HBsAg, regimen administered (entecavir/tenofovir), and HBV viral load as independent variables (Table 2). In this model, using optimal scaling CATREG SPSS algorithm, FIB-4 was discretized in 5 categories: (i) ≤ 1.16 (Quantification factor −0.923); (ii) 1.17–2.89 (Quantification factor 0.205); (iii) 2.90–4.27 (Quantification factor 1.334); (iv) 4.28–6.97 (Quantification factor 2.462); (v) ≥6.98 (Quantification factor 3.591). The results showed that HBV pgRNA was not independently correlated with FIB-4 (*p* = 0.137) after adjustment for (AST)1/2, (AST/ALT)1/2, 1/PLT, age, sex, HBsAg, HBV viral load, regimen administered, and duration of therapy (ordinal regression ANOVA *p* < 10–12; R2reg: 0.794) (Figure 3).

**Table 2:** Categorical regression model assessing the potential value of HBV pgRNA as prognosticator of FIB-4 using categorical regression after using maximal discretization, ridge regression regularization, and 10× cross-validation.

**Variables**	**Optimal binary cutoff**	**Beta**	**SE(beta) estimate**	***F***	***p***	**Tolerance**
AGE^†^	63	0.203	0.048	17.947	< 0.001	0.704
(AST/ALT)^1/2‡^	0.98	0.291	0.042	49.051	< 0.001	0.690
(AST)^1/2¶^	4.47	0.398	0.066	36.629	< 0.001	0.600
1/PLT^§^	4.46	0.355	0.062	33.158	< 0.001	0.851
HBV pgRNA	>0	0.086	0.057	2.264	0.137	0.542
Male gender	NA	0.041	0.031	1.678	0.199	0.826
Duration of therapy	126	−0.015	0.039	0.157	0.693	0.741
HBsAg	1,100	−0.015	0.041	0.126	0.724	0.678
Regimen	NA	0.007	0.024	0.073	0.788	0.855
HBV viral load	1,000	−0.011	0.044	0.063	0.802	0.668

^†^Quantification factors for “Age” (7 categories): −1.939 (≤38 years), −1.305 (39–47 years), −0.670 (48–55 years), −0.036 (56–62 years), 0.598 (63–69 years), 1.233 (70–80 years), 1.867 (≥81 years).

^‡^Quantification factors for “(AST/ALT)^1/2^” (6 categories): −1.265 (≤0.86); −0.598 (0.87–0.98); 0.068 (0.99–1.09); 0.735 (1.10–1.20); 1.401 (1.21–1.37); 2.068 (≥1.38).

^¶^Quantification factors for “(AST) ^1/2^” (3 categories): −1.118 (≤4.46), 0.230 (4.47–24.01); 4.273 (≥24.02).

^§^Quantification factors for “1/PLT” (6 categories): −1.352 (≤3.44); −0.622 (3.45–4.45); 0.108 (4.46–5.42); 0.838 (5.43–6.48); 1.568 (6.49–8.12); 2.298 (≥8.13).

NA, not applicable.

### 3.5 HBV pgRNA and PAGE-B

**Figure 4:**
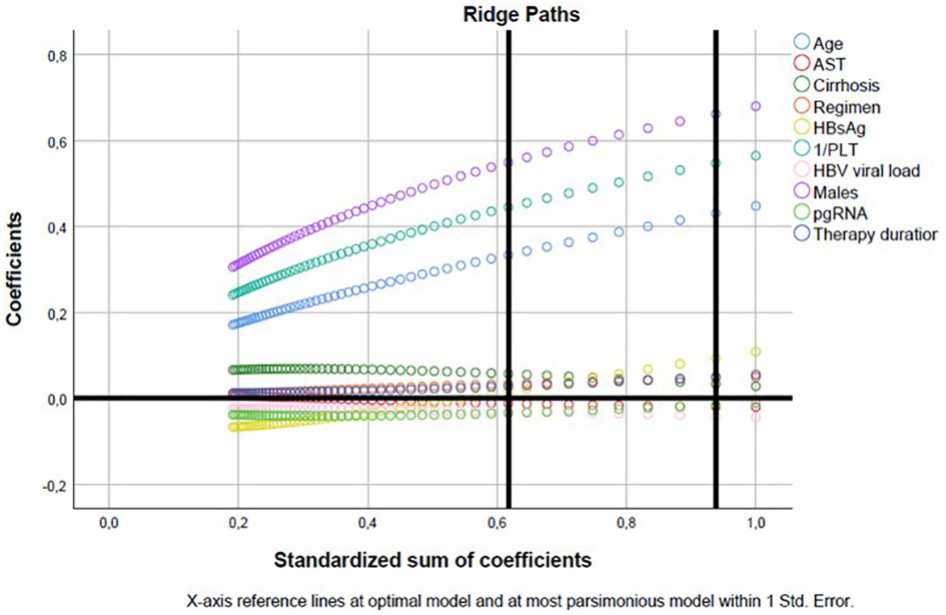
Categorical regression model assessing the potential value of HBV pgRNA as prognosticator of PAGE-B (Table 3); ridge paths.

To further investigate the potential role of HBV pgRNA title as an independent prognosticator for the PAGE-B score, we implemented a multivariable categorical regression model incorporating the latter as the dependent variable and age, sex, platelets, duration of therapy, presence of cirrhosis, HBV pgRNA, male sex, regimen administered, HBsAg, and HBV viral load as independent variables. Similarly, using the optimal scaling CATREG SPSS algorithm, PAGE-B was discretized into seven *categories:* (i) ≤ 6 (quantification factor −1.524); (ii) 8–9 (Quantification factor −1.012); (iii) 10–11 (Quantification factor −0.500); (iv) 12–13 (Quantification factor 0.012); (v) 14–15 (Quantification factor 0.523); (vi) 16 (quantification factor 1.035); (vii) ≥17 (quantification factor 1.547) (Figure 4). Notably, HBV pgRNA was not correlated with PAGE-B (*p* = 0.459) after adjustment for age, sex, AST, 1/PLT, duration of therapy, HBsAg, HBV viral load, regimen administered, and presence of cirrhosis (ordinal regression ANOVA *p* < 10–12; R2reg: 0.800) (Table 3).

**Table 3:** Categorical regression model assessing the potential value of HBV pgRNA as prognosticator of PAGE-B using categorical regression after using maximal discretization, ridge regression regularization, and 10× cross-validation.

**Variables**	**Optimal binary cutoff**	**Beta**	**SE(beta) estimate**	***F***	***p***	**Tolerance**
AGE^†^	63	0.334	0.047	49.425	< 0.001	0.686
Male gender^‡^	NA	0.549	0.039	194.206	< 0.001	0.843
1/PLT^¶^	4.46	0.445	0.043	106.215	< 0.001	0.848
Duration of therapy	126	0.028	0.047	0.367	0.546	0.736
HBV pgRNA	>0	−0.034	0.046	0.554	0.459	0.566
Cirrhosis	NA	0.057	0.036	2.528	0.116	0.762
AST	25	−0.012	0.045	0.071	0.790	0.701
Regimen	NA	0.034	0.033	1.042	0.310	0.855
HBsAg	1,100	0.015	0.047	0.106	0.745	0.689
HBV viral load	1,000	−0.030	0.036	0.723	0.398	0.723

^†^Quantification factors for “Age” (7 categories): −1.939 (≤ 38 years), −1.305 (39–47 years), −0.670 (48–55 years), −0.036 (56–62 years), 0.598 (63–69 years), 1.233 (70–80 years), 1.867 (≥81 years).

^‡^Quantification factors for “Male gender” (2 categories): −0.978 (0: female.86); 1.023 (1: male).

^¶^Quantification factors for “1/PLT” (6 categories): −1.352 (≤3.44); −0.622 (3.45–4.45); 0.108 (4.46–5.42); 0.838 (5.43–6.48); 1.568 (6.49–8.12); 2.298 (≥8.13).

NA: not applicable.

## 4 Discussion

The presence of both CP-direct and preC RNA in HBeAg-negative HBe antibody-positive CHB patients has been studied. We observed low pgRNA positivity rates in our study group, indicating fluctuating levels among the long-term NA-treated individuals. Accordingly, despite the relatively low pgRNA positivity rates in our study group, we found that pgRNA levels were not correlated with either the fibrosis marker (FIB-4) or the HCC predictive marker PAGE-B. However, it was strongly and negatively correlated with the NA treatment duration.

HBV has a complicated life cycle, and cccDNA is its transcriptional template and a means of replication and self-preservation. Given the genomic complexity of the virus, little is known about the circulation of HBV RNA. HBV RNA circulation varies according to the illness stage, NAs therapy, and HBV genotype (22). Among the nine different genotypes, HBV resembled many of the mutants. Genotype D dominates globally and is associated with various nucleoside substitutions in core and pre-core promoters (23). All the subjects tested for the HBV genotype were found to be positive for subtype D. In most cases, G1869A, a missense mutation, is present at a high frequency in the Greek population and is highly related to the HBV D genotype and absence of e antigen (3). Mutations affect various elements of gene expression, levels of HBV RNA circulation, replication and transcriptional activity of cccDNA. Considering studies on HBV RNA levels in serum, only a few describe a fluctuation between pgRNA and preC RNA levels, while in others, no preC RNA was found in serum (24, 25). The presence of HBe Ag is more likely to C and B genotypes, where the presence of HBV pgRNA seems to be positively correlated with HBV DNA load and HbS Ag even after a 5-year NAs therapy duration (26, 27). Given our small patient population we have shown that the HBV pgRNA prevalence is statistically differentiate among our patients. In fact there is a significant fluctuation among the patients treated with NAs for less than a year and those treated more than a year, without to exceed 6 years, (*p-value* = 0.021 < 0.05). Generally, HBeAg positive genotypes seem to be more virulent and have a straightforward correlation between the HBe Ag levels and the HBV pgRNA detectability (28). However, our cohort lacks patients with other HBV genotypes except for D, which had already mentioned, so we cannot conclude about the relation between those two viral indexes.

In our data, there was an absence of preC RNA, which justifies the prevalence of HBeAg-negative virions. This phenomenon is explained by various mutations harbored in the core promoter, such as A1762T, G1764A, and C1653T, which decrease preC RNA levels and e-antigen secretion. Another factor that could explain the absence of preC RNA in our samples is the significantly low levels of pgRNA (median 2.89; 2.51–3.44, log10 copies/ml), which are usually elevated compared to preC RNA (29). Moreover, our patients were at least 6 months under NA treatment, resulting in low detection of pgRNA and HBV DNA.

In a study by Gahny et al. (30) a significant correlation was demonstrated between high levels of HBV RNA (*p* < 0.0001) and FIB-4, indicating that HBV pgRNA detectability could sufficiently predict both FIB-4 staging and advanced liver fibrosis. In contrast, we demonstrated that there is no independent correlation between HBV pgRNA and FIB-4 after adjustment for FIB-4 components (namely (AST)1/2, (AST/ALT)1/2, 1/PLT, age), sex, HBsAg, HBV viral load, regimen administered, and duration of therapy. This immediately indicates that pgRNA has no value as a potential prognosticator of FIB-4. However, the difference regarding the HBV pgRNA correlation with the FIB-4 score could be addressed to our small cohort of 88 total individuals. At the same time, the study of Gahny et al. had much larger patient group reaching 1,409 participants. In addition, the presentage of HBV pgRNA-positive individuals was three times above from ours (58% compared to 18.1%). Perhaps, such major differences in sampling could be compared, if only our sampling was scalable to more individuals.

Furthermore, we investigated the possible correlation between HBV pgRNA and HCC predictability using a non-invasive scoring system. The PAGE-B is a score that predicts the 5-year cumulative HCC incidence with 0% for those scoring ≤ 9, 3% for 10–17 and 17% for those scoring ≥18. Based on our results, the multivariable model applied to the assessment of HBV pgRNA as an independent variable did not show any correlation with the PAGE-B score; hence, pgRNA had no prognostic value as far as PAGE-B was concerned.

Even if our findings are not persuaded as for the use of HBV pgRNA as a prominent biomarker in relation with the already existed prognostic scores. We highly encourage its correlation with other molecular markers, which seem to add up predictive value on the liver fibrotic status. Such a combinational study seems to set in spotlight malecular biomarkers related to hepatocytes' proliferation migration and even apoptosis (31, 32). For instance, Ding et al. discuss the poorer overall survival of HCC patients with undetectable HBV DNA levels under a longterm NA tretment, who found positive with high HBV pgRNA levels. Most of all they compared the HBV pgRNA detectability with the overexression of insulin-like growth factor 2 mRNA-binding protein 3 (IGF2BP3), an oncoprotein that provides hepatocellular microenvironment with stemness (33).

NAs are a successful treatment for eliminating HBV DNA load. HBV pgRNA was negatively correlated with NAs treatment duration (Spearman *r* = –0.4335*, p* < 0.0001) (Figure 1B). In addition, 18.1% of our subjects tested positive for HBV pgRNA, indicating possible cccDNA transcriptional activity even in HBV DNA-negative patients (31%). Wang et.al agreed with our findings depicting 63.64% of HBV pgRNA-positive among the HBV DNA-negative group. This finding suggests that although NAs are a successful treatment, rapidly eliminating HBV load and HBV pgRNA could be useful in preventing premature therapy cessation (34, 35). This assumption is supported by findings suggesting that NAs discontinuation after 5 years of treatment leads to illness relapse in patients with detectable HBV pgRNA levels (13, 14).

### 4.1 Limitations

Our study has limitations that we would like to mention. Initially, we have a small group of patients with pgRNA positive. Consequently, we could not perform analysis with different genotype of HBV, due to the prevalence of D genotype in our study population. Lastly, we were unable to assess pgRNA in HBe Ag positive patients, because we lack patients with this serological profile in our region. Hence, the next essential goal is to analyze our results in a larger group of patients with different serological profiles.

## Data Availability

The raw data supporting the conclusions of this article will be made available by the authors, without undue reservation.
