# Supplementary material for: Is there any role for HBV pgRNA in fibrosis and HCC predisposition?
Source: Front Med (Lausanne). 2025 Nov 12;12:1678116. doi: 10.3389/fmed.2025.1678116 (PMC12647073; doi:10.3389/fmed.2025.1678116)
Supplement: Supplementary file 1 [file Data_Sheet_1.DOCX]

Supplementary Material

# Supplementary Data

Supplementary Material should be uploaded separately on submission. Please include any supplementary data, figures and/or tables.

Supplementary material is not typeset so please ensure that all information is clearly presented, the appropriate caption is included in the file and not in the manuscript, and that the style conforms to the rest of the article.

# Supplementary Figures and Tables

For more information on Supplementary Material and for details on the different file types accepted, please see [here](https://www.frontiersin.org/guidelines/author-guidelines#supplementary-material).

## Supplementary Figures


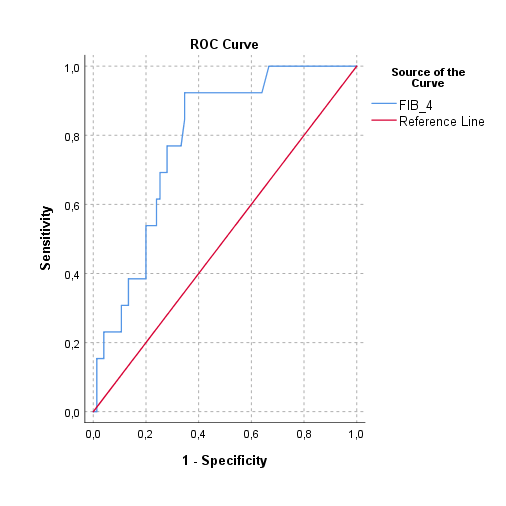


**Supplementary Figure 1**. The use of FIB-4 score to assess Fibroscan documented cirrhosis; AUROC curve: 0.783 ± 0.057 (95 CI: 0.671 – 0.895; p = 8x10-7; overall model quality: 0.67)

## Supplementary Tables

**Supplementary Table 1.**Custom made RT-PCR sequences for the quantitative determination of HBV RNA

|  | **RT-PCR Sequences** |
| --- | --- |
| Forward Primer PCP | 5′-GGTCTGCGCACCAGCACC-3′ |
| Forward Primer PGP | 5′-CACCTCTGCCTAATCATC-3′ |
| Forward Promer M3 | 5′-CTGGGAGGAGTTGGGGGAGGAGATT-3′ |
| Reverse Primer BC1 | 5′-GGAAAGAAGTCAGAAGGCAA-3’ |
| Probe hbvLC | 5′-TGGAGGCTTGAACAGTAGGACATGAAC-3′ |
| Probe hbvFL | 5′-CYAAAGCCACCCAAGGCACAGC-3′ |

**Supplementary Table 2.**Cloned plasmid sequence of HBV cccDNA

|  | **RT-PCR Sequences** |
| --- | --- |
| Cloned plasmid sequence | 5′-CTGGGAGGAGTTGGGGGAGGAGATTAGGTTAAAGGTCTTTGTACTAGGAGGCTGTAGGCATAAATTGGTCTGCGCACCAGCACCATGCAACTTTTTCACCTCTGCCTAATCATCTCTTGTTCATGTCCTACTGTTCAAGCCTCCAAGCTGTGCCTTGGGTGGCTTTGGGGCATGGACATCGACCCTTATAAAGAATTTGGAGCTACTGTGGAGTTACTCTCGTTTTTGCCTTCTGACTTCTTTCC -3′ |
